# Atherogenic index of plasma is an effective index for estimating abdominal obesity

**DOI:** 10.1186/s12944-018-0656-1

**Published:** 2018-01-15

**Authors:** Shi-Wei Shen, Yun Lu, Feng Li, Cheng-Jian Yang, Yin-Bo Feng, Hong-Wei Li, Wei-Feng Yao, Zhen-Hai Shen

**Affiliations:** 1 Wuxi No.2 People’s Hospital Affiliated to Nanjing Medical University, Wuxi, Jiangsu 214002 China; 2 The Taihu Rehabilitation Hospital of Jiangsu Province (Jiangsu Provincial Research Center for Health Assessment and Intervention), Wuxi, Jiangsu 214086 China

**Keywords:** Atherogenic index of plasma (AIP), Waist circumference, Abdominal obesity

## Abstract

**Background:**

The correlation between the atherogenic index of plasma (AIP) and waist circumference (WC) remains unknown.

**Methods:**

A total of 5351 middle-aged men living in Southeastern China were surveyed using the random stratified cluster sampling method. A WC of 90 cm or greater was indicative of abdominal obesity, and AIP was calculated as follows: log [triglyceride (TG)/high-density lipoprotein-cholesterol (HDL-C)].

**Results:**

(1) A significantly higher AIP was observed in subjects with abdominal obesity than in those without abdominal obesity (*P* < 0·001). (2) Multivariate logistic regression analysis revealed an odds ratio of 1·93, 2·59 and 2·76 for abnormal AIP levels for the second, third and fourth WC quartiles, respectively (all *P* < 0·001) compared to the first WC quartile as a reference. (3) There was a linear correlation between WC and AIP, and a 1·0 cm increase in WC resulted in a 0·0175 rise in AIP. For AIP corresponding to moderate risk (0·12–0·21), WC was 85–90 cm; for AIP corresponding to high risk (> 0·21), WC was >90 cm.

**Conclusions:**

AIP of 0·12–0·21 or >0·21 indicates a likelihood of borderline abdominal obesity or abdominal obesity, respectively, and the combination of WC and AIP may increase the specificity and sensitivity for detection of abdominal obesity in clinical practice. The results suggest that AIP may be used as a reference to estimate abdominal obesity.

## Background

Obesity has become an important public health concern worldwide, and abdominal obesity, which involves fat accumulation in the abdomen, is recognized as an independent risk factor for obesity-related diseases and death [1]. In China, obesity has become a major risk factor for the increased prevalence of cardiovascular diseases [2]. In the present study, waist circumference (WC) was used as an index to assess abdominal obesity in middle-aged men, and the correlation between WC and the atherogenic index of plasma (AIP) was evaluated to provide evidence for the development of a preventive and control strategy for atherosclerosis and cardiovascular diseases in community populations.

## Methods

**Fig. 1:**
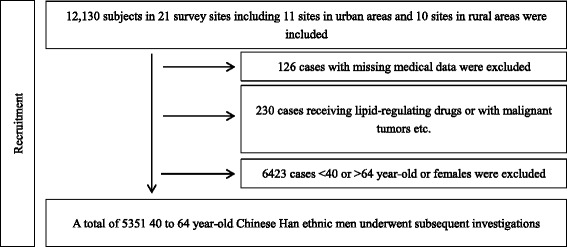
Participant Flowchat

A total of 21 survey sites (11 sites in urban areas and 10 sites in rural areas) were sampled in the three cities of Suzhou, Wuxi and Changzhou, Jiangsu Province, China, during the period from January to December 2009 using the random stratified cluster sampling method. Three villages were randomly sampled from each survey site, and approximately 200 permanent adult residents (duration of residence >5 years) were randomly sampled from each village. A total of 12,130 residents were investigated, of whom 11,774 had complete medical data available and were enrolled in the analysis. A total of 5351 men of Chinese Han ethnicity and aged 40–64 years underwent subsequent investigations, including 2810 (52·51%) living in urban areas and 2541 (47·49%) living in rural areas. Those receiving lipid-regulating drugs or with malignant tumors, Cushing’s syndrome, a history of schistosome infections, severe hepatic and renal insufficiency, or incomplete medical records were excluded from this study. (Fig. 1).

The study protocol was approved by the Ethics Review Committee of our hospital, and signed informed consent was obtained from all participants.

All data were collected using a questionnaire designed by medical professionals. Health examinations included height, body weight, WC, blood pressure measurements, and blood lipid and glucose levels. Body mass index (BMI) was calculated using the following formula: BMI = body weight (kg)/height^2^ (m^2^). WC was measured using the method recommended by the World Health Organization. In brief, each subject was instructed to stand with the feet 25–30 cm apart and with the back straight to allow even distribution of body weight on two legs, and then the distance between the anterior superior iliac spine and the midpoint of the inferior margin of the 12th rib was measured at the end of a normal expiration. For biochemical examinations, all participants fasted for 8–12 h before collection of 5 mL of venous blood from the cubital vein the following morning. Levels of total cholesterol (TC), low-density lipoprotein-cholesterol (LDL-C), high-density lipoprotein-cholesterol (HDL-C), triglyceride (TG) and fasting blood glucose (FBG) were determined using the oxidase method, a homogeneous assay, an antibody-based homogeneous assay, the glycerol phosphate oxidase method, and the hexokinase method, respectively, on a fully automatic Hitachi 7180 biochemical analyzer (Hitachi, Tokyo, Japan).

### Diagnostic criteria

According to the International Diabetes Federation 2005 criteria [3], abdominal obesity was defined as WC of ≥ 90 cm for men. AIP was calculated as log (TG/HDL-C) [4]. AIP risk was assigned into three groups: (1) low risk, AIP ≤ 0·11; (2) moderate risk, AIP ≥ 0·12 and ≤ 0·21; and (3) high risk, AIP > 0·21 [5]. Obesity was defined as BMI of ≥ 28 kg/m^2^ [6] and hypertension as systolic blood pressure (SBP) of ≥ 140 mmHg and/or diastolic blood pressure (DBP) of ≥ 90 mmHg, or currently undergoing antihypertensive therapy. Hyperglycemia was defined as FBG of >6·1 mmol/L or currently receiving antidiabetic agents, and hyperuricemia as serum uric acid (UA) of ≥ 420 mmol/L [7]. Hypertriglyceridemia was defined as TG of >1·7 mmol/L and hypercholesterolemia as TC of >5·18 mmol/L, while a HDL-C concentration of <1·04  mmol/L indicated low HDL-C and a concentration of >3·37 mmol/L indicated high LDL-C [8].

### Statistical analysis

All measurement data are expressed as mean ± standard deviation, and all statistical analyses were performed using the statistical software SPSS version 11.5 (SPSS, Chicago, IL, USA). Differences in means among groups were tested for statistical significance using the chi-square test, and associations between WC and cardiovascular risk factors were examined using multivariate logistic regression analysis and linear correlation analysis. A *P* value <0·05 was considered statistically significant.

## Results

### Comparison of cardiovascular risk factors in subjects stratified by age and WC

Higher mean AIP, BMI, UA, SBP, DBP, TG, TC, LDL-C and FBG were observed in subjects with abdominal obesity compared to those with normal WC (all *P* < 0·001). However, no significant difference was seen in TC level between subjects with abdominal obesity and those with normal WC aged 60–64 years (*P* > 0·05). In addition, a lower mean HDL-C concentration was noted in subjects with abdominal obesity than in those with normal WC (*P* < 0·001) (Table 1).

**Table 1:** Comparison of cardiovascular risk factors in subjects stratified by age groups and WC (χ ± S)

Age(years)	WC (cm)	*n*	BMI(kg/m^2^)	TG(mmol/L)	TC(mmol/L)	HDL-c(mmol/L)	LDL-c(mmol/L)	UA(μmol/L)	FBG(mmol/L)	SBP(mmHg)	DBP(mmHg)	AIP
40–44	<90	764	23.22 ± 2.45	1.92 ± 1.63	4.66 ± 0.85	1.24 ± 0.36	2.87 ± 1.14	374.76 ± 72.72	5.32 ± 0.79	124.7 ± 13.31	75.7 ± 11.09	0.12 ± 0.31
	≥90	590	27.05 ± 2.53	2.69 ± 2	4.88 ± 0.88	1.12 ± 0.25	3.21 ± 0.91	405.85 ± 76.77	5.79 ± 1.59	133.84 ± 15.25	82.64 ± 11.03	0.31 ± 0.31
	*P* Value		0.00	0.00	0.00	0.00	0.00	0.00	0.00	0.00	0.00	0.00
45–49	<90	689	23.22 ± 2.22	1.88 ± 1.58	4.78 ± 0.86	1.25 ± 0.35	2.93 ± 0.89	364.98 ± 70.34	5.44 ± 1.05	125.74 ± 13.95	77.15 ± 10.62	0.11 ± 0.31
	≥90	661	26.96 ± 2.32	2.8 ± 2.16	4.94 ± 0.9	1.14 ± 0.31	3.27 ± 0.91	408.91 ± 82.37	5.91 ± 1.41	135.2 ± 16.03	83.54 ± 10.96	0.32 ± 0.32
	*P* Value		0.00	0.00	0.00	0.00	0.00	0.00	0.00	0.00	0.00	0.00
50–54	<90	587	23.44 ± 2.09	1.74 ± 1.36	4.62 ± 0.88	1.26 ± 0.32	2.82 ± 0.82	366.16 ± 69.02	5.61 ± 1.39	127.43 ± 16.5	77.71 ± 11.12	0.08 ± 0.3
	≥90	538	26.98 ± 2.45	2.45 ± 1.78	4.82 ± 0.94	1.16 ± 0.44	3.17 ± 0.9	399.92 ± 83.27	6.09 ± 1.62	135.37 ± 15.99	83.52 ± 10.6	0.26 ± 0.31
	*P* Value		0.00	0.00	0.00	0.00	0.00	0.00	0.00	0.00	0.00	0.00
55–59	<90	467	23.58 ± 2.34	1.65 ± 1.28	4.64 ± 0.86	1.28 ± 0.29	2.79 ± 0.86	365.68 ± 73.55	5.65 ± 1.26	130.7 ± 17.54	78.63 ± 11.05	0.04 ± 0.3
	≥90	499	26.89 ± 2.25	2.39 ± 2.02	4.82 ± 0.92	1.16 ± 0.37	3.14 ± 0.93	400.89 ± 78.69	6.12 ± 1.5	137.04 ± 17.85	82.93 ± 11.09	0.25 ± 0.31
	*P* Value		0.00	0.00	0.00	0.00	0.00	0.00	0.00	0.00	0.00	0.00
60–64	<90	275	23.57 ± 2.19	1.69 ± 1.12	4.72 ± 0.77	1.27 ± 0.40	2.89 ± 0.83	367.46 ± 76.37	5.7 ± 1.03	134.29 ± 18.14	79.07 ± 11.57	0.06 ± 0.3
	≥90	281	27.01 ± 2.26	2.25 ± 1.94	4.83 ± 0.89	1.17 ± 0.24	3.11 ± 0.89	391.6 ± 89.05	6.29 ± 1.78	141.99 ± 17.84	83.22 ± 10.37	0.21 ± 0.32
	*P* Value		0.00	0.00	0.10	0.00	0.00	0.00	0.00	0.00	0.00	0.00
Total	<90	2782	23.36 ± 2.28	1.80 ± 1.47	4.68 ± 0.86	1.26 ± 0.42	2.86 ± 0.94	368.28 ± 71.95	5.50 ± 1.11	127.49 ± 15.72	77.31 ± 11.08	0.09 ± 0.31
	≥90	2569	26.98 ± 2.38	2.56 ± 2.01	4.86 ± 0.91	1.15 ± 0.40	3.19 ± 0.91	402.88 ± 81.48	6.00 ± 1.56	136.02 ± 16.58	83.17 ± 10.86	0.28 ± 0.32
	Total	5351	25.10 ± 2.95	2.17 ± 1.79	4.77 ± 0.89	1.21 ± 0.41	3.02 ± 0.94	384.89 ± 78.59	5.74 ± 1.37	131.59 ± 16.69	80.12 ± 11.36	0.18 ± 0.33
	P Value		0.00	0.00	0.00	0.00	0.00	0.00	0.00	0.00	0.00	0.00

### Pearson correlation analysis of WC and cardiovascular risk factors

Pearson correlation analysis revealed that WC was positively correlated with AIP (*r* = 0·371), BMI (*r* = 0·786), UA (*r* = 0·283), SBP (*r* = 0·241), DBP (*r* = 0·224), TG (*r* = 0·266), TC (*r* = 0·139), LDL-C (*r* = 0·243) and FBG (*r* = 0·215) (all *P* < 0·001), and negatively correlated with HDL-C (*r* = −0·222) (*P* < 0·001).

### Multivariate logistic regression analysis of various WC quartiles and cardiovascular risk factors

All subjects were assigned to WC quartiles: (1) first quartile (Q1), WC ≤ 84 cm; (2) second quartile (Q2), WC ≥ 85 and ≤ 89 cm; (3) third quartile (Q3), WC ≥ 90 and ≤ 94 cm; and (4) fourth quartile (Q4), WC ≥ 95 cm. After adjustment for age, SBP, DBP, BMI, UA, TC, LDL-C and FBG, multivariate logistic regression analysis revealed an odds ratio of 1·93, 2·59 and 2·76 for abnormal AIP levels in Q2, Q3 and Q4 for WC (all *P* < 0·001) with WC Q1 as the reference (Table 2).

**Table 2:** Multivariate logistic regression analysis of various quartiles of WC and cardiovascular risk factors

WC group	N	Regression Coefficient	Wald chi square value	OR(95%CI)	*P*值
Q1(WC ≤ 84 cm)	1463			1.00	
Q2(WC85-89 cm)	1319	0.66	27.40	1.93 (1.51–2.46)	0.00
Q3(WC90-94 cm)	1326	0.95	61.90	2.59 (2.05–3.29)	0.00
Q4(WC ≥ 95 cm)	1243	1.02	62.83	2.76 (2.15–3.55)	0.00

Notes: Age, SBP, DBP, BMI, UA, TC, LDL-C and FBG were adjusted

### Detection rates of abdominal obesity in various AIP quartiles

The chi-square test revealed an increase in the detection rate of abdominal obesity with increasing AIP quartile (*P* < 0·001): the detection rate of abdominal obesity was 2·52 times greater in AIP Q4 than in AIP Q1 (Table 3).

**Table 3:** Detection rates of abdominal obesity in various quartiles of AIP

AIP groups	n(WC ≥ 90 cm)	Detection Rates(%)	Chi square value	*P* value
Q1(≤ − 0.050)	1337 (352)	26.31	600.831	0.000
Q2(>-0.050 ≤ 0.133)	1338 (594)	44.42	33.210	0.000
Q3(>0.133 ≤ 0.389)	1339 (737)	55.04	27.222	0.000
Q4(>0.390)	1337 (886)	66.26	283.059	0.000
Total	5351 (2569)	48.01	16.957	0.000

Note: Linear-by-Linear Association 456.202, *P* = 0.000

### Linear regression analysis of WC and mean AIP

**Fig. 2:**
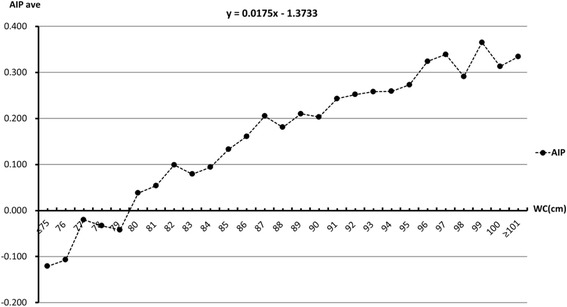
Linear regression analysis of WC and mean AIP

WC was classified into 27 groups (group 1, WC ≤ 75 cm; Groups 2–26, WC = 76–100 cm; and group 27, WC ≥101 cm) and the mean AIP was calculated in each group. Linear regression analysis revealed a linear correlation between WC and AIP; a 1·0 cm increase in WC resulted in a 0·0175 rise in AIP (Fig. 2).

## Discussion

Abdominal obesity, also known as central obesity, is mainly characterized by the deposition of fat in the subcutaneous abdominal region and perivisceral region. It has been shown that excessive accumulation of body fat may cause an increase in the prevalence of multiple cardiovascular risk factors, including hyperinsulinemia, insulin resistance, hypertension and dyslipidemia, which are considered the major causes of obesity-induced cardiovascular diseases [9]. We found a significantly higher AIP in subjects with abdominal obesity than in those without abdominal obesity (*t* = −22·276, *P* < 0·001), and WC was positively correlated with BMI, AIP, UA, SBP, DBP, TG, TC, LDL-C and FBG, and negatively correlated with HDL-C. In addition, we found that AIP increases with WC, and a rise in AIP resulted in an increase in the detection rate of abdominal obesity. These results demonstrate that WC and AIP are parameters associated with lipid metabolism, and an increase in WC is indicative of abnormal deposition of visceral fat in the abdomen, while a rise in AIP is indicative of dyslipidemia.

Results from the Action to Control Cardiovascular Risk in Diabetes (ACCORD) study showed that among patients undergoing treatment with statins alone, the incidence of primary endpoint events was greater in those with high TG and low HDL-C levels than in other patients [10]. However, Pietro Scicchitano et al. [11] proposed functional food and nutraceuticals have the potential to become the future of primary prevention in dyslipidaemia treatment in cardiovascular disease prevention. Therefore, management of atherogenic dyslipidemia, which is characterized by hypertriglyceridemia and low HDL-C concentrations, should be a focus of attention [12].

It has been found that AIP is negatively associated with LDL particle diameter [4], and an increase in AIP indicates a rise in the proportion of small dense LDL (sdLDL) [13]. Relative to LDL, sdLDL is more likely to be oxidized and promote the production of foam cells, and sdLDL is therefore accepted as a strong risk factor for atherosclerosis and a predictive factor for emergency cardiovascular events [14]. In 2002, sdLDL was identified as a major risk factor for coronary heart disease by the National Cholesterol Education Program, and its detection was recommended by the program [15]. However, all currently available approaches used to detect sdLDL have limitations and are difficult to popularize in clinical practice. By contrast, measurement of AIP is simple, economical and feasible. Although AIP is a calculated value, it is a sensitive indicator of dyslipidemia, and may indirectly reflect the diameter of LDL-C particles [13]. We therefore hypothesized that the combination of WC and AIP may increase the specificity and sensitivity of the detection of abdominal obesity in clinical practice.

We observed a linear correlation between WC and AIP, and a 1·0 cm increase in WC resulted in a 0·0175 increase in AIP. When WC increased from 84 to 90 cm, AIP increased from 0·094 to 0·203, and AIP was ≥ 0·243 when WC was ≥ 91 cm, indicating that WC was ≤ 84, 85–90 and >91 cm for AIP corresponding to low (≤ 0·11), moderate (0·12–0·21) and high risk (> 0·21), respectively. These results demonstrate that moderate-risk AIP indicates borderline abdominal obesity, and high-risk AIP indicates the presence of abdominal obesity.

Although WC intuitively reflects abdominal fat accumulation, it fails to quantify and differentiate visceral fat and subcutaneous fat. Considering the limitations and inconvenience of computed tomography and magnetic resonance imaging in clinical practice, a simpler way to identify abdominal obesity is of great significance for the prevention and control of cardiovascular diseases in community populations. The WC cutoff point for abdominal obesity remains controversial. The World Health Organization and International Diabetes Federation recommend WC ≥ 90 cm for men and ≥80 cm for women as the cutoff points for abdominal obesity in the Asian Pacific region [3, 16]. The Working Group on Obesity in China recommends WC ≥ 85 cm for men and ≥ 80 cm for women as the cutoff points for abdominal obesity [6], while the 2011 Chinese Guidelines for Prevention of Cardiovascular Diseases recommend WC cutoff points of ≥ 90 cm for men and ≥ 85 cm for women for abdominal obesity [8]. In addition, the Japan Society for the Study of Obesity recommends WC cutoff points of 85 cm for men and 90 cm for women [17], and 85 cm was predicted as the WC cutoff point for insulin resistance in middle-aged Japanese men [18]. He and colleagues reported WC cutoff points of 83–85 cm for men and 73–76 cm for women in China [19], and our recent study showed that an appropriate WC cutoff point was 85 cm for abdominal obesity in middle-aged men living in Suzhou, Wuxi and Changzhou areas of Jiangsu Province, southeastern China [20]. In the present study we found that WC was 85–90 cm when AIP was 0·12–0·21, which strongly supports the previous results. Therefore, we suggest that WC cutoff points for abdominal obesity should be defined according to local epidemiological profiles.

Our study has some limitations. First, computed tomography or magnetic resonance imaging was not performed to quantitatively differentiate visceral fat and subcutaneous fat. Second, the effects of race, gender, age, region and economic levels on moderate- and high-risk AIP levels require further investigation.

## Conclusion

In conclusion, moderate- or high-risk AIP indicates a likelihood of borderline abdominal obesity or abdominal obesity, respectively, and the combination of WC and AIP may increase the specificity and sensitivity of the detection of abdominal obesity in clinical practice. Our results suggest that AIP may be used as a reference to identify abdominal obesity.

## Abbreviations

AIP: Atherogenic index of plasma
BMI: Body mass index
DBP: Diastolic blood pressure
FBG: Fasting blood glucose
HDL-C: High-density lipoprotein-cholesterol
LDL-C: Low-density lipoprotein-cholesterol
SBP: Systolic blood pressure
TC: Total cholesterol,
TG: Triglyceride
UA: Uric acid
WC: Waist circumference
